# Ethnic variance on long term clinical outcomes of concomitant use of proton pump inhibitors and clopidogrel in patients with stent implantation: A PRISMA-complaint systematic review with meta-analysis

**DOI:** 10.1097/MD.0000000000024366

**Published:** 2021-02-12

**Authors:** Wence Shi, Lu Yan, Jingang Yang, Mengyue Yu

**Affiliations:** a State Key Laboratory of Cardiovascular Disease; b Fuwai Hospital, National Center for Cardiovascular Diseases, Chinese Academy of Medical Sciences and Peking Union Medical College, Beijing, China.

**Keywords:** clopidogrel, comedication, ethnicity, proton pump inhibitors, stent implantation

## Abstract

**Background:**

Pharmacokinetic and pharmacodynamic study showed a lower clopidogrel response when coprescribed with proton pump inhibitors (PPIs). Despite this, PPIs is necessary for patients treated with long term dual antiplatelet therapy (DAPT). Ethnic variance also played a different effect on clopidogrel response. Our study evaluated the effect of concomitant use of DAPT and PPIs and assessed whether ethnic variance exert different effect on clinical outcomes.

**Methods:**

We carefully searched EMBASE, PubMed/Medline databases, and the Cochrane library in April 2019. The primary endpoint was major adverse cardiovascular and cerebrovascular events (MACCE) and individual endpoints reported. We also focused on bleeding events. Studies were excluded if the follow-up were <12 months and patients were not treated with clopidogrel after stent implantation.

**Results:**

A total of 18 studies were included in the systematic review (involving 79,670 patients). No randomized controlled trials (RCTs) were included. PPIs comedication were associated with increased MACCE (odds ratio [OR] = 1.38; 95% confidence interval [CI] = 1.28–1.49) while not associated with decreased bleeding risks, such as gastrointestinal bleeding (OR = 1.05; 95% CI = 0.53–2.11). PPIs comedication were associated with increased risk for all endpoints among Caucasian population while not with increased risk for MACE (OR = 1.20; 95% CI = 0.99–1.39), all-cause death (OR = 1.24; 95% CI = 0.74–2.06), cardiac-death (OR = 1.29; 95% CI = 0.64–2.57) among Asian population.

**Conclusion:**

PPIs comedication were associated with adverse clinical outcomes, and ethnic variance may exert different effect on clinical outcomes. Subgroup analysis indicated that concomitant use of PPI might be suitable for Asian patients after stent implantation.

## Abstract 2

Supplemental Digital Content is available in the text

## 1 Introduction

The crucial role played by clopidogrel in preventing ischemic events have been fully demonstrated in the past 2 decades and clinical guidelines recommended that clopidogrel should be given for at least 12 months after stent implantation.^[1]^ Extended antiplatelet agents decrease the risk for major adverse cardiovascular and cerebrovascular events (MACCE), very late stent thrombosis, and myocardial infarction,^[2]^ while risk for upper and lower gastrointestinal bleeding (GIB) increased.^[3]^ Therefore proton pump inhibitors (PPIs) are often comedicated to protect gastrointestinal (GI) tract. However, studies showed a potential drug-interaction which would attenuate clopidogrel antiplatelet function and result in adverse clinical outcomes.^[4]^

Although, the European Society of Cardiology and the European Association for Cardio-Thoracic Surgery (ESC/EACTS) in 2018 stated that routine PPI comedication with dual antiplatelet therapy (DAPT) is not recommended,^[5]^ routine PPIs use seem popular reported in some studies. Furthermore, CYP2C19 allele which encode the key enzyme in the metabolism of clopidogrel differs among ethnics^[6,7]^ which may exert a different effect on clinical outcomes among different ethnics. Therefore, we tried to make this meta-analysis to explore these problems.

## 2 Methods

This meta-analysis was designed according to the Preferred Reporting Items for Systematic Reviews and Meta-Analyses (PRISMA) guidelines. (Supplement PRISMA checklist, http://links.lww.com/MD/F670).

### 2.1 Data sources and search strategy

Two reviewers (W-CS, LY) carefully searched EMBASE, PubMed/Medline databases, and the Cochrane library for Randomized Controlled Trials (RCTs) and observational studies. We used “proton pump inhibitor,” “clopidogrel,” “percutaneous coronary intervention,” “stent implantation” as key words to search in databases. Additionally, their abbreviation such as PPI, PCI, and DAPT were also used. In order to search more studies, we widen the key words and “omeprazole,” “pantoprazole,” “lansoprazole,” “esomeprazole,” “rabeprazole,” and “thienopyridine” were included in our search strategies.

### 2.2 Criteria for inclusion/exclusion

Original, research studies published or presented to April 2019 in English were eligible for inclusion. Studies comparing PPIs comedication with clopidogrel alone in patients after stent implantation were included. Population without definite stent implantation and clopidogrel medication were excluded. Follow-up <12 months and adverse cardiovascular outcomes (especially major adverse cardiovascular disease) were not reported as their clinical endpoints were also ineligible. Conference abstracts and studies where full articles could not be retrieved were not initially excluded from the search strategy but later excluded from the meta-analysis

### 2.3 Study selection

We imported identified studies into NoteExpress and duplicates were deleted. Two investigators (SWC and YL) independently screened all titles and abstracts returned by the search strategy. Then 2 investigators (SWC and YL) viewed all full text copies of potential relatively studies. A third investigator (YMY) resolved any discordance in assessments.

### 2.4 Study endpoints

The primary clinical endpoints chosen for this analysis was MACCE and individual endpoints reported such as all-cause death (ACD), cardiac death (CD), myocardial infarction (MI), stroke, stent thrombosis (ST), target vessel revascularization (TVR), and target lesion revascularization (TLR). We also reported safety endpoints including bleeding events and net adverse clinical events (NACE).

### 2.5 Data extraction

Two investigators (SWC and YL) independently used a standardized data form (supported by Microsoft Excel) to extracted study characteristics (author, study design, country), total number of patients, type of PPI, type of clinical endpoints, coronary risk factors, and follow-up. We extract data with propensity score matching (PSM) in priority if available.^[8,9]^ Disagreements regarding the appropriateness of studies included for analysis were resolved by discussion and consultation with a third investigators of our group (YMY).

### 2.6 Risk of bias assessment

Two investigators (SWC and YL) assessed risk of bias, and a third investigator (YMY) resolved discrepancies by consensus. The New-castle-Ottawa Scale was used to assess the methodological quality of observational studies in terms of validating participant selection, population comparability, and outcome/exposure assessment. (Supplement 1, http://links.lww.com/MD/F606 and Supplement 2, http://links.lww.com/MD/F607). Only one post hoc analysis of randomized trial^[10]^ was included and we used recommendations from the Cochrane Collaboration.

### 2.7 Data synthesis and analysis

Measures of association, including odds ratios (ORs), hazard ratios (HRs), and relative risks (RRs), with 95% confidence intervals (CIs), were extracted from included studies. We pooled adjusted ORs, HRs, and RRs because adverse cardiovascular events were rare. Review Manager version 5.3 for Windows was utilized to analyze the weighted mean, variance of the overall effect, 95% CI and *P*-value, and generate forest plots for exposure-outcome comparisons in each dataset. Separate analyses of primary endpoint and individual endpoints were prespecified. Considering the inherent differences between these study designs, a fixed effects model (*I*^2^ < 50%) or a random effects model (*I*^2^ > 50%) was used based on the value of *I*^2^ obtained and also a sensitivity analysis was conducted by individual exclusion of each study for each outcome to assess their effects on the pooled outcome. OR with 95% CIs were calculated and we measured significance using a *P* value of <.05. Publication bias was assessed by observing funnel plots.

### 2.8 Ethical review

Our manuscript is a pooled analysis of former published articles and no ethical approval is applicable.

## 3 Results

### 3.1 Study selection

**Figure 1:**
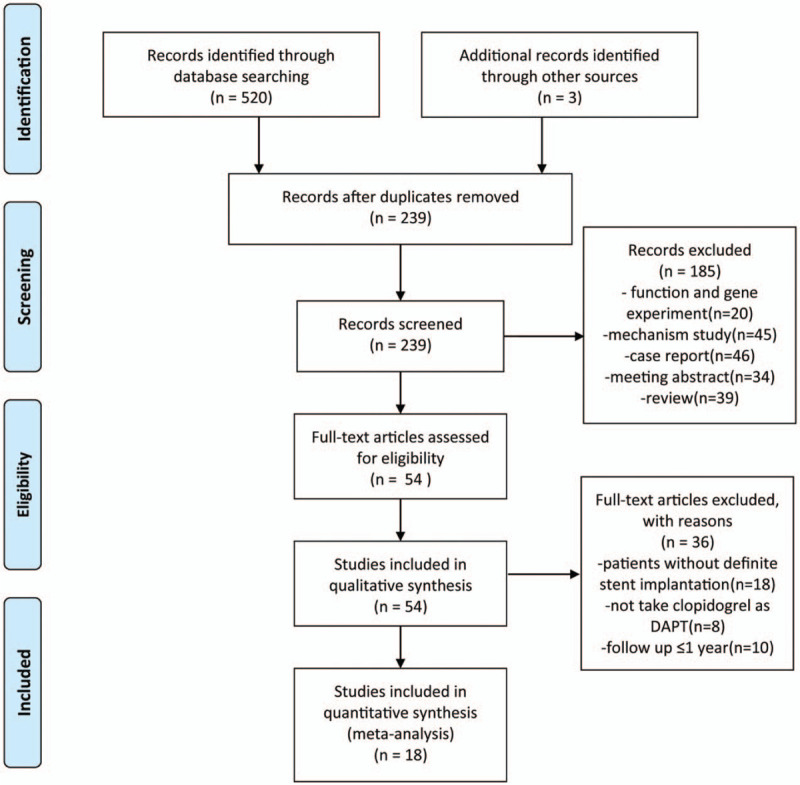
Flow diagram for the study selection.

A total of 523 articles were download after researching the databases by keywords mentioned above and there are 239 articles remained after removing duplicates. One hundred eighty five articles were excluded for reasons such as function and gene experiment, mechanism studies, case report, or review. Another 36 articles did not meet the including criteria we set for this study. Therefore we pooled 17 cohort studies^[8,9,11–25]^ and one post-hoc analysis of RCT^[10]^ together in the following meta-analysis finally (Fig. 1).

### 3.2 Population and baseline characteristics

A total of 79,670 patients (31,732 patients treated with clopidogrel plus PPIs and 47,938 patients treated with clopidogrel alone) aged 60 years or older were available for analyses. All of the patients take aspirin (75–100 mg) and clopidogrel as DAPT routinely recommended in guidelines. Six studies were conducted in Asian country (China^[8,10]^ and Japan^[9,17,18,25]^), others were conducted in Europe and America such as US,^[11,12,20,22,23]^ Italy,^[11,12,20,22,23]^ Austria,^[19]^ Greece,^[24]^ and 3 multi-country studies.^[13,15,16]^ The PPIs used in these studies included esomeprazole, omeprazole, pantoprazole rabeprazole, and lansoprazole (Table 1).

**Table 1:** Studies included in present article.

Study/Year	Location	Study design	Patients	No. of subjects PPI(+): PPI(−)	Age (years) PPI(+): PPI(−)	DM (%) PPI(+): PPI(−)	HTN (%) PPI(+): PPI(−)	Hyperlipidemia (%) PPI(+): PPI(−)	Smoking (%) PPI(+): PPI(−)	Aspirin dose, mg/d	PPI studied	Follow-up	MACE (composite outcome definition)	Individual outcomes reported
Pei Zhu/2017	China	Cohort	PCI	1966:1966	60.2 ± 10.6:57.7 ± 10.3	29.0: 30.3	64.0: 65.4	66.9: 71.7	NM	100	NM	2-year	ACD, MI, TVR, ST, stroke	ACD,MI, TVR, ST, stroke, bleeding, BARC 3 or 5, GI bleeding
Jaya/2017	US/EU	Cohort	PCI with stent	1062:3573	64.1 ± 11.4: 65.4 ± 11.1	33.0: 32.8	79.8: 80.4	77.5: 75.3	16.9: 20.2	NM	NM	2-year	CD, ST, MI, TLR	CD, ST, MI, TLR, BARC 3 or 5, NACE
Jackson/2016	US	Cohort	AMI with PCI	1636:7196	63 (55–70):59 (51–67)	32.4: 25.2	76.1: 64.8	73.1: 63.9	NM	NM	NM	1-year	Death, MI, TVR, stroke	GUSTO moderate/severe bleeding
Yan Y/2016	Multi	Cohort	ACS with PCI	4814:4126	66.22 (56.39–73.80) 61.25 (52.00–71.00)	26.1: 22.4	59.5: 49.6	48.4: 42.0	NM	NM	NM	1-year	ACD, re-infarction	ACD, re-infarction, bleeding (GUSTO moderate/severe bleeding)
Gargiulo G/2016	Italia	Cohort	PCI	375:612	71.8 (63.8–77.7) 67.9 (58.9–74.5)	23.3: 24.8	72.5: 71.3	53.8: 55.3	NM	75-100	NM	2-year	ACD, MI, CVA	ACD, CD, MI, ST, BARC 3 or 5, GUSTO moderate/severe bleeding, NACE
Weisz /2015	Multi	Cohort	PCI with DES	2162:6419	64.4 ± 10.5 63.2 ± 11.0	34.8: 31.4	83.7: 77.8	76.9: 73.2	10.4: 6.5	NM	NM	2-year	CD, MI, TLR	CD, MI, TVR, ST, bleeding
Zou J/2014	China	Post hoc analysis	PCI with DES	6188:1456	66.2 ± 10.2 65.7 ± 10.6	25.8: 23.6	71.3: 70.4	60.2: 62.3	32.2: 31.0	100	NM	1-year	Death, MI, TVR, TLR, CABG, ST	ST, MI, death, TVR, TLR, CABG
Burkard K/2012	NM	Cohort	PCI with stent	109:692	66.5 ± 10.563.3 ± 11.3	29.6: 17.2	72.5: 65.0	73.4: 75.9	24.8: 29.8	100	O, E, P, L	3-year	CD, MI, TVR	CD, MI, TVR, ST
Chitose /2012	Japan	Cohort	PCI	187:443	69.7 ± 11.4: 69.6 ± 10.6	35.3: 33.7	77.9:79.0	61.9: 61.7	23.9: 26.2	100	NM	18-month	CD, MI, stroke	CD, MI, stroke, GI event
Aihara H/2012	Japan	Cohort	PCI	500:500	69 ± 11:68 ± 10	40.8: 39.4	71.2: 69.0	83.0: 83.8	44.6: 43.2	100	NM	3-year	ACD, MI	ACD, MI, stroke, ST, GI bleeding
Kimura T/2011	Japan	Cohort	PCI with stent	3223:9223	69.0 ± 11.267.7 ± 10.9	39: 37	83: 82	NM	31: 32	≥81	O, R, L	3-year	CD. MI, stroke	CD, MI, stroke, ST, GUSTO moderate/severe bleeding, GI bleeding
Rossini R/2011	Italy	Cohort	PCI with DES	1158:170	64 ± 11 63 ± 11	27.1: 28.0	63.6: 65.2	65.8: 72.5	49.3: 49.7	100	L, O, P	1-year	Death, MI, destabilizing symptoms leading to hospitalization, and nonfatal stroke	Death, ST
Tentzeris/2010	Austria	Cohort	PCI with stent	691:591	64.11 ± 12.42:64.44 ± 11.87	18.7: 26.0	73.7: 78.2	76.4: 77.1	27.9: 23.1	100	NM	1-year	ACD, ACS, ST	ACD, CD, ACS, ST,
Kreutz/2010	US	Cohort	PCI with stent	6828:9862	67.5 ± 10.4:65.2 ± 10.6	25.9: 22.7	50.6: 46.5	67.8: 63.4	NM	NM	O, E, P, L, R	1-year	Stroke, TIA, ACS, CD, CABG, PCI	Stroke or TIA, MI or UA, CABG, PCI, CD, MI, UA, PCI, CABG
Gaglia/2010	US	Cohort	PCI with DES	318:502	63.8 ± 11.663.7 ± 11.6	36.3: 33.1	78.5: 74.8	87.7: 82.4	13.8: 18.9	325	E, L, O, P, R	1-year	ACD, MI, TVR, ST	ACD, MI, TVR, ST
Gupta/2010	US	Cohort	PCI	72:243	61.7 ± 1.262 ± 0.7	36: 30	76: 68	67: 60	25: 33	NM	NM	4-year	Death, MI, target vessel failure	Death, TLR, target vessel failure
Zairis M/2010	Greece	Cohort	PCI with stent	340:248	62.1 ± 10.5 61.7 ± 10.8	30.0: 26.2	50.9: 46.4	66.5: 65.3	49.7: 50.8	100-325	O	1-year	CD, MI	CD, MI, ST,
Yasu/2010	Japan	Cohort	PCI with DES	188:103	69.0 ± 9.667.4 ± 10.1	35.0: 39.7	64.1: 64.8	68.0: 57.8	24.3: 27.1	NM	R	395-day	CD, ACS, ST, TLR	CD, ACS, ST, TLR

ACD = any-cause death, ACS = acute coronary syndrome, BARC 3 or 5 = bleeding academic research consortium 3 or 5, DES = drug eluting stents, DM = diabetes mellitus, E = esomeprazole, GI bleeding = gastrointestinal bleeding, HTN = hypertension, L = lansoprazole, MI = myocardial infarction, NM = not mentioned, O = omeprazole, P = pantoprazole, PCI = percutaneous coronary intervention, R = rebeprazole, ST = stent thrombosis, TLR = target lesion revascularization, TVR =  target vessel revascularization.

### 3.3 Risk of bias assessment

All 18 studies included for meta-analysis showed good over-all methodological quality. The descriptions of population selection, exposure, and outcome ascertainment are clear mentioned. Most studies had a high competence of follow-up with outcome data. However, few studies used prescription and pharmacy dispensing record databases to ascertain exposure and current procedural terminology fourth edition (CPT-4) codes.^[20]^ In addition, choice of antiplatelet therapy and PPIs use were left to the discretion of the individual treating physicians in accordance with practice guideline recommendations and local standards of care in all studies.

### 3.4 Heterogeneity assessment

We found no heterogeneity among MACCE (*X*^2^ = 29.10, *P* = .03; *I*^2^ = 42%), ACD (*X*^2^ = 11.52, *P* = .12; *I*^2^ = 39%), MI (*X*^2^ = 18.79, *P* = .07; *I*^2^ = 41%), ST (*X*^2^ = 4.71, *P* = .97; *I*^2^ = 0%), TVR (*X*^2^ = 7.95, *P* = .16; *I*^2^ = 37%), TLR (*X*^2^ = 6.16, *P* = .10; *I*^2^ = 51%), and bleeding events except gastrointestinal bleeding (*X*^2^ = 11.25, *P* = .01; *I*^2^ = 73%). However statistically significant heterogeneity were observed in CD (*X*^2^ = 28.29, *P* = .005; *I*^2^ = 58%), and stroke (*X*^2^ = 16.50, *P* = .002; *I*^2^ = 76%).

### 3.5 MACCE and individual outcomes

**Figure 2:**
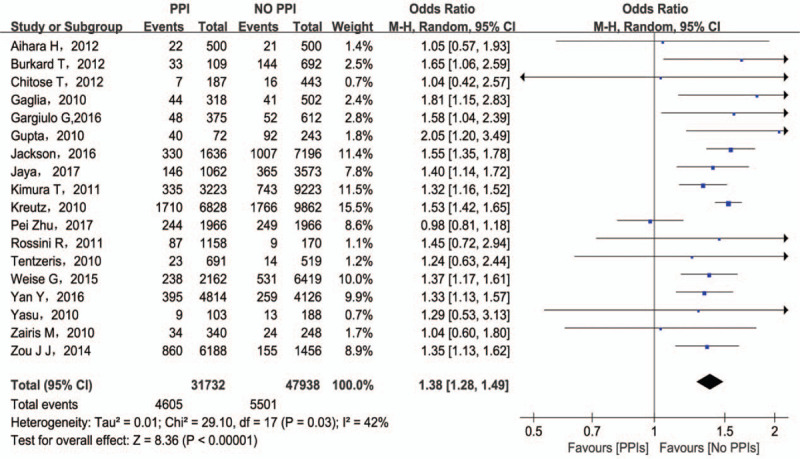
MACCE associated with the concomitant use of clopidogrel and PPIs. MACCE = major adverse cardiovascular and cerebrovascular events; PPI = proton pump inhibitors.

**Figure 3:**
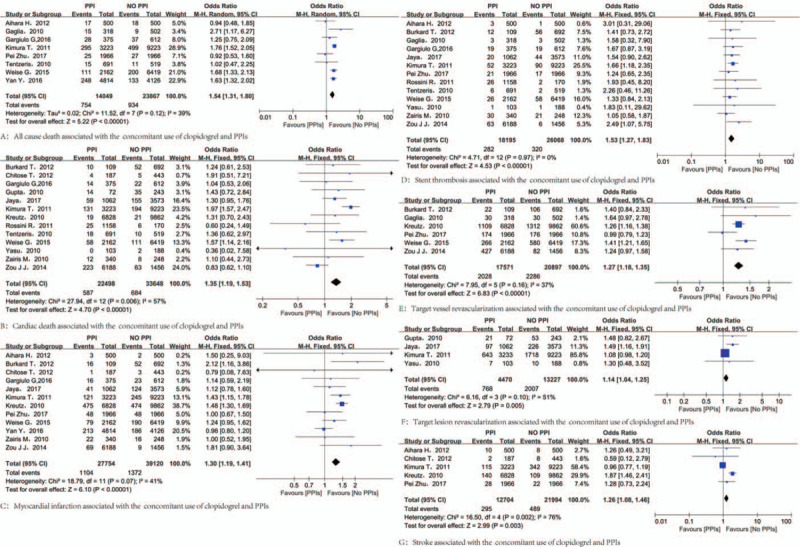
Results for individual endpoints of MACCE. MACCE = major adverse cardiovascular and cerebrovascular events.

Concomitant therapy showed a statistically significant increase in composite MACCE compared with clopidogrel monotherapy (OR = 1.38; 95% CI = 1.28–1.49) (Fig. 2**)**. Although the definition of MACCE were divergent in different studies, as for individual components of MACCE, concomitant therapy were associated with increased risk for ACD (OR = 1.54; 95% CI = 1.31–1.80), CD (OR = 1.35; 95% CI = 1.19–1.53), MI (OR = 1.30; 95% CI = 1.19–1.41), ST (OR = 1.53; 95% CI = 1.27–1.83), TVR (OR = 1.27; 95% CI = 1.18–1.35), TLR (OR = 1.14; 95% CI = 1.04–1.25), and stroke (OR = 1.26; 95% CI = 1.08–1.46) (Fig. 3).

### 3.6 Bleeding outcomes

**Figure 4:**
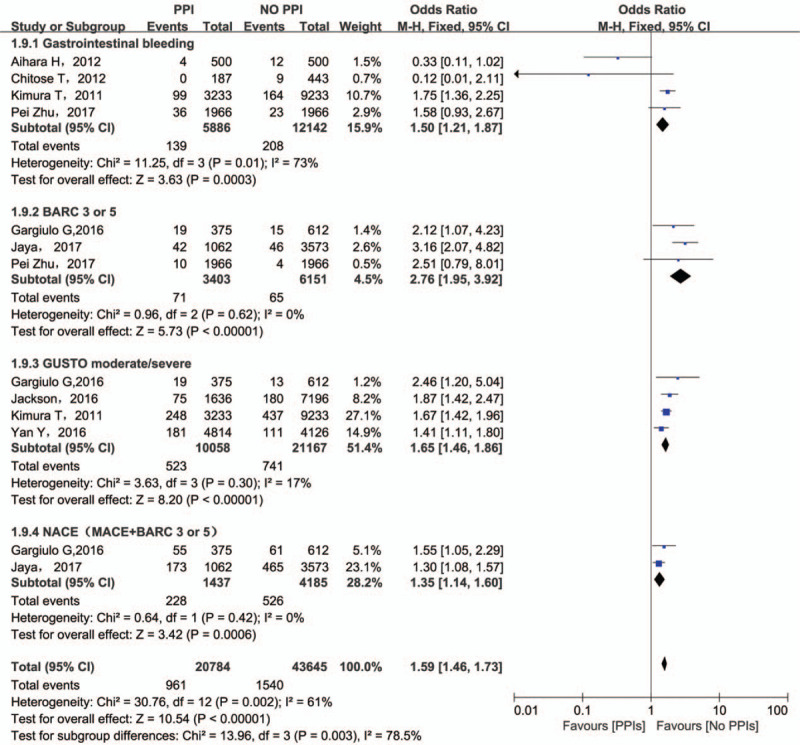
Results for bleeding events and NACE. NACE = net adverse clinical events.

The main purpose of combined treatment is to prevent GI bleeding. However, the use of PPIs did not decrease the risk for GIB (OR = 1.50; 95% CI = 1.21–1.87) (Fig. 4). Although a significant heterogeneity was found among these 4 studies reporting GIB, only exclusion of Kimura (2011)^[11]^ changed the results indicating that PPIs medication had no effect on GIB (OR = 1.00; 95% CI = 0.65–1.55). The differences in baseline clinical characteristics among these studies are responsible for the results. As for BARC 3/5 bleeding and GUSTO moderate/severe bleeding events, PPIs are related to bleeding events with OR of 2.80 (95% CI = 1.98–3.96) and OR of 1.66 (95% CI = 1.44–1.91) respectively (Fig. 4).

### 3.7 NACE

Net adverse clinical event (NACE) could evaluate comprehensive effect of concomitant therapy which is defined as a composite endpoint including bleeding events and MACCE. Only 2 studies report NACE and the pooled analysis is in favor of clopidogrel monotherapy (OR = 1.35; 95% CI = 1.13–1.60). (Fig. 4).

### 3.8 Subgroup analysis

**Figure 5:**
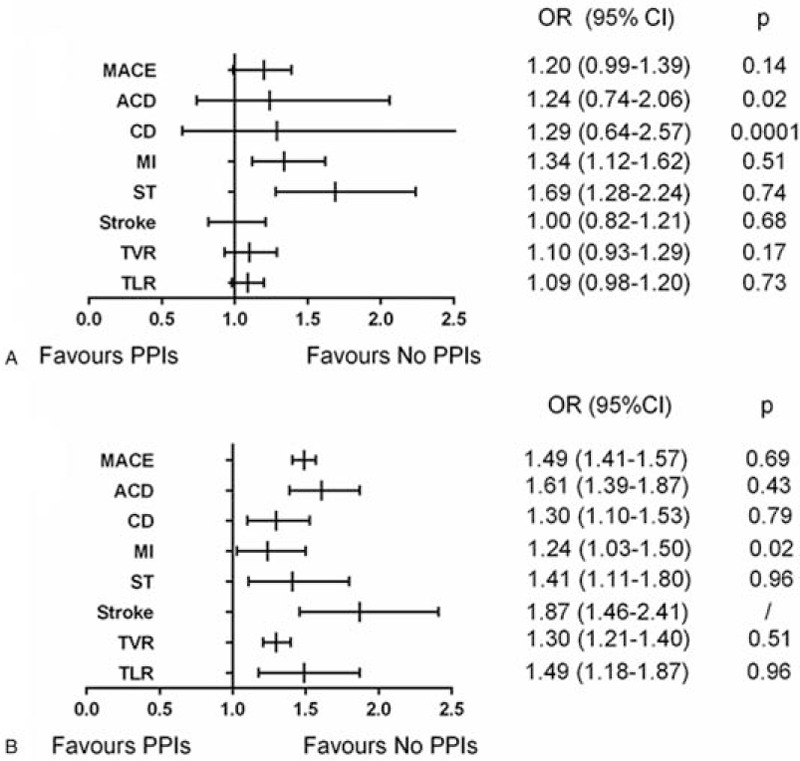
Pooled results for Asian population (A) and Caucasian population (B).

We made a subgroup analysis considering the discrepancy in ethnics in order to illuminate the potential difference between Caucasians and Asian population. We found Asian patients treated with PPIs concurrently showed no significant difference in MACE (OR = 1.20; 95% CI = 0.99–1.39), ACD (OR = 1.24; 95% CI = 0.74–2.06), CD (OR = 1.29; 95% CI = 0.64–2.57), stroke (OR = 1.00; 95% CI = 0.82–1.21), TVR (OR = 1.10; 95% CI = 0.93–1.29), and TLR (OR = 1.09; 95% CI = 0.98–1.20). (Fig. 5A) However, the results in Caucasians population support the monotherapy of PPI for all endpoints (Fig. 5B).

### 3.9 Sensitivity analysis

In a sensitivity analysis by single study exclusion for each outcome, Kimura (2011) cause the heterogeneity among CD, GI, and stroke. However, the results did not change while the exclusion of Kimura (2011). In Caucasians population during the subgroup analysis, Kreutz (2010)^[20]^ resulted the heterogeneity in MI and changed the outcome opposite to the concomitant use (OR = 1.11; 95% CI = 0.96–1.27). For other outcomes, the exclusion of any study did not significantly alter the results or the heterogeneity.

## 4 Discussion

In this meta-analysis pooling 18 studies, we found no benefits in all clinical endpoints for patients with PPIs comedication. But a divergent result after subgroup analysis according to ethnics was identified.

Clopidogrel is a prodrug that depends on cytochrome P450 (CYP) with isoenzyme CYP2C19 playing a major role to generate an active metabolite,^[26]^ PPIs also interact with the CYP, which may inhibit the conversion of clopidogrel to its active metabolite and potentially alter its antiplatelet properties.^[4]^ Some pharmacokinetic experiments show an interaction between PPIs and clopidogrel which would attenuate its antiplatelet effect.^[27–30]^ In addition, recent study has demonstrated that patients with reduced-function CYP2C19 allele lead to reduced levels of active clopidogrel metabolites, which are associated with worse cardiovascular outcomes, including stent thrombosis.^[30]^ In our study, the results seemed support the theory.

Moreover, several mechanisms impairing endothelial function have been reported to account for the complications of PPI use.^[31]^ Dimethylarginine dimethylamino-hydrolase (DDAH) is present in all cells, degrading asymmetric dimethylarginine (ADMA), which inhibits the endothelial enzyme nitric oxide synthase (eNOS). While PPI use would inhibit DDAH and increase ADMA, this would reduce levels of vasodilator nitric oxide (NO). Vascular NO inhibits thrombosis and vascular inflammation.^[32]^ Therefore, PPI use was associated with a broad impairment in endothelial function which would be expected to increase major adverse cardiovascular events.

A series studies demonstrate a hypofunctional CYP2C19 metabolic phenotypes variance from 13% to 23% in healthy East Asian populations to only 2% to 5% in Caucasians.^[7]^ Therefore we made a subgroup analysis to demonstrate the ethnic variance on the effect of concomitant use. The results showed the concomitant use did not bring statistically adverse effect among Asian population on MACE, ACD, CD, stroke, TVR, and TLR, but the effect on MI, ST still remained. It seemed that patients with hypofunctional CYP2C19 metabolic phenotypes might benefit more from DAPT-PPIs combination therapy. In China, physicians prescribed DAPT (especially clopidogrel as first-line medicine) to patients after stent implantation, and study reported that GIB incidence is higher in Chinese AMI population.^[33,34]^ Therefore the PPIs usage is prevalent in China. But we found no study focused on this topic and Chinese physicians tend to prescribe PPIs on recommendation from guideline based on studies from Caucasians population. Our study indicated that the adverse effect of concomitant use seemed less in Asian population. In addition, a small recent RCT show that prescription of PPI was associated with higher compliance with DAPT and decreases the risk of recurrent cardiovascular events.^[35]^ Therefore Asian population might benefit from concomitant use to some degree. However, the definite mechanism and effect of ethnic variation on this topic still unknown, further pharmacokinetic and pharmacodynamic studies, and largescale clinical trials especially RCTs were warranted.

In addition, PPIs were analysis in our study as a class. Several studies indicated that PPIs are metabolized by CYP2C19, but to a varying degree.^[36]^ Clopidogrel response were measured by VASP in Thomas study^[37]^ showed that patients receiving pantoprazole had a significantly better platelet response to clopidogrel compared with omeprazole. Another study focused on esomeprazole and rabeprazole demonstrated no association with impaired response to clopidogrel by testing VASP.^[38]^ However, the most suitable PPI for Asians is still unclear and more prospective studies are warranted.

Overall, ethnic variation on concomitant therapy indicated that a lower threshold for PPIs prescription might be suitable in Asian population. What's more, our research provides a perspective for future research that ethnic differences are potential factors that may affect drug metabolism and clinical outcomes, and we should increase our concern on it.

### 4.1 Limitations

Several limitations in this meta-analysis affect the conclusions. No randomized trials included may affected the results and because of this reason, the bias risk of the studies was not assessed using recommendations from the Cochrane Collaboration. The definition and standard for diagnose for MACCE, ST, and GIB are different among included studies which may potentially affect the conclusion. Few studies focus on the bleeding endpoints such as GI bleeding and NACE, and subgroup analysis were not made in these endpoints, therefore the ethnic variance on bleeding events and NACE were short in this study. PPI used in different studies were analyzed as a class in our meta-analysis, and PPIs with varied inhibition of CYP2C19 might exert different clinical effect. Because the choice of PPI use was left to the discretion of the individual physicians in involved studies, it's hard to make separate analysis showing differential response with ethnic variance.

## 5 Conclusions

The present systematic review and meta-analysis found consistent evidence of an association between concomitant drug-use and adverse clinical outcomes, we also identified an ethnic variance on clinical outcomes. The results suggested that prescription for PPIs among Asian patients may suitable. New evidence focus on ethnic variance are warranted.

## Acknowledgments

The authors thank all co-authors, especially YJG and YMY who have supported this manuscript.

## Author contributions

**Conceptualization:** Mengyue Yu.

**Data curation:** Wence Shi, Lu Yan.

**Formal analysis:** Wence Shi.

**Funding acquisition:** Mengyue Yu.

**Investigation:** Lu Yan.

**Methodology:** Lu Yan, Jingang Yang.

**Project administration:** Jingang Yang, Mengyue Yu.

**Software:** Wence Shi, Lu Yan.

**Supervision:** Lu Yan, Mengyue Yu.

**Validation:** Mengyue Yu.

**Writing – original draft:** Wence Shi.

**Writing – review & editing:** Jingang Yang, Mengyue Yu.

## Abbreviations

Abbreviations: ACD = all-cause death, ACS = acute coronary syndrome, BARC 3 or 5 = bleeding academic research consortium 3 or 5, DES = drug eluting stents, E = esomeprazole, GI bleeding = gastrointestinal bleeding, L = lansoprazole, MI = myocardial infarction, NM = not mentioned, O = omeprazole, P = pantoprazole, PCI = percutaneous coronary intervention, R = rebeprazole, ST = stent thrombosis, TLR = target lesion revascularization, TVR = target vessel revascularization.
